# Statistically correcting dynamical electron scattering improves the refinement of protein nanocrystals, including charge refinement of coordinated metals

**DOI:** 10.1107/S2059798320014540

**Published:** 2021-01-01

**Authors:** Thorsten B. Blum, Dominique Housset, Max T. B. Clabbers, Eric van Genderen, Maria Bacia-Verloop, Ulrich Zander, Andrew A. McCarthy, Guy Schoehn, Wai Li Ling, Jan Pieter Abrahams

**Affiliations:** a Department of Biology and Chemistry, Paul Scherrer Institute, 5232 Villigen, Switzerland; b Université Grenoble Alpes, CEA, CNRS, IBS, 71 Avenue des Martyrs, 38000 Grenoble, France; c Center for Cellular Imaging and NanoAnalytics (C-CINA), Biozentrum, University of Basel, Mattenstrasse 26, 4058 Basel, Switzerland; d European Molecular Biology Laboratory, 71 Avenue des Martyrs, 38042 Grenoble, France

**Keywords:** electron diffraction, dynamical scattering, protein, nanocrystals, likelihood-based correction

## Abstract

Electron diffraction allows protein structure determination when only nanosized crystals are available. Nevertheless, multiple elastic (or dynamical) scattering, which is prominent in electron diffraction, is a concern. Current methods for modeling dynamical scattering by multi-slice or Bloch wave approaches are not suitable for protein crystals because they are not designed to cope with large molecules. Here, dynamical scattering of nanocrystals of insulin, thermolysin and thaumatin was limited by collecting data from thin crystals. To accurately measure the weak diffraction signal from the few unit cells in the thin crystals, a low-noise hybrid pixel Timepix electron-counting detector was used. The remaining dynamical component was further reduced in refinement using a likelihood-based correction, which was introduced previously for analyzing electron diffraction data of small-molecule nanocrystals and was adapted here for protein crystals. The procedure is shown to notably improve the structural refinement, in one case allowing the location of solvent molecules. It also allowed refinement of the charge states of bound metal atoms, an important element in protein function, through *B*-factor analysis of the metal atoms and their ligands. These results clearly increase the value of macromolecular electron crystallography as a complementary structural biology technique.

## Abstract 2

A likelihood-based method is improved to correct for dynamical scattering in weakly diffracting protein nanocrystals. The method has been tested on three protein crystals and yielded better refinement statistics and structure interpretations, including charge estimations of bound ions.

## 1. Introduction   

The strong interaction of electrons with matter favors electron crystallography for solving three-dimensional structures from beam-sensitive sub-micrometre-sized crystals such as protein nanocrystals (Clabbers & Abrahams, 2018 ▸; Henderson, 1995 ▸). Using cryo-electron microscopy, protein crystals can be vitrified and studied in their native hydrated states. In contrast to real-space techniques such as single-particle analysis or electron tomography, electron diffraction data are recorded in the diffraction plane. The major advantage of image-based methods over diffraction is that they provide experimental data with crystallographic phases. However, compared with diffraction methods, this advantage comes at a severe price in terms of signal strength, as the electron dose required for a single high-resolution still image is sufficient for a full 3D diffraction rotation data set from the same crystal (Clabbers & Abrahams, 2018 ▸). Indeed, various protein structures have recently been solved using diffraction data collected with continuous rotation of 3D crystals (Clabbers *et al.*, 2017 ▸; de la Cruz *et al.*, 2017 ▸; Nannenga *et al.*, 2014 ▸; Xu *et al.*, 2018 ▸; Yonekura *et al.*, 2015 ▸).

Early attempts to use electron diffraction in structural biology were promising but were mainly limited by the electron detectors. Coupled with phase information obtained from images, structures were solved to 6–7 Å resolution in some early studies (Subramaniam *et al.*, 1997 ▸; Unwin & Henderson, 1975 ▸). Only a few still diffraction patterns could be recorded from any single (2D) protein crystal with the imaging plates or charge-coupled detectors used in these studies. The limitation is due to the significant radiation damage that is imposed on the crystals by the dose that is necessary to obtain sufficient signal strength using these low-sensitivity detectors. The recent development of direct electron detectors has drastically reduced the dose required to achieve a data quality suitable for data analysis.

In parallel, another important technological advancement was achieved in CMOS and hybrid pixel detectors, which allow low-noise and high-speed imaging at high dynamic ranges, which is especially important for diffraction studies. Owing to the very low curvature of the Ewald sphere for diffraction in typical transmission electron microscopes operating in the 100–300 kV range, still diffraction patterns yield only quasi-2D planes in diffraction space. Fast detectors allow the 3D sampling of reciprocal space by collecting diffraction data from continuously rotating samples and the straightforward integration of the reflections that are fully recorded during the rotation (Gemmi *et al.*, 2019 ▸; van Genderen *et al.*, 2016 ▸).

Electron diffraction data collection with fast sensitive electron detectors from continuously rotating crystals has yielded data with a quality and quantity that permits standard X-ray crystallography programs such as *MOSFLM* (Leslie, 2006 ▸), *XDS* (Kabsch, 2010 ▸) or *DIALS* (Clabbers *et al.*, 2018 ▸) to process the electron diffraction data. Data integration became straightforward using a range of mature public domain software packages, each with a wide user base and supported by longstanding crystallographic software consortia. There is also a range of standard crystallographic software for subsequent structure solution by molecular replacement or direct methods (if permitted by the resolution) and refinement, for example the *CCP*4 suite (Winn *et al.*, 2011 ▸) and *SHELX* (Usón & Sheldrick, 1999 ▸).

Nevertheless, such programs do not consider multiple elastic (dynamical) scattering. Dynamical scattering is negligible in X-ray diffraction, but this is not the case in electron diffraction owing to the strong interaction of electrons with matter. As multiple elastic scattering angles coincide with kinematic Bragg angles, the measured intensities can no longer be simply approximated as the squares of the structure factors. Dynamical diffraction theory has been successfully used for dynamical structure refinement of electron diffraction data (Palatinus *et al.*, 2017 ▸). However, such methods require knowledge of the atomic structure, the shape and the orientation of the crystal to model the electron wavefunction traveling through the crystal. Currently, such an approach is only feasible for small molecules with small unit cells.

Data-collection strategies may be used to help to minimize multiple scattering events in macromolecular crystals. In particular, thin crystals can be selected to reduce the scattering path length. Recent results have shown that microscopic crystals can also be thinned using a focused ion beam to a specified thickness for electron diffraction experiments (Duyvesteyn *et al.*, 2018 ▸; Li *et al.*, 2018 ▸; Martynowycz *et al.*, 2019 ▸; Zhou *et al.*, 2019 ▸; Beale *et al.*, 2020 ▸). A data-collection protocol in which the crystal is continuously rotated about a random axis will reduce dynamical scattering through the integration (and thus averaging) of Bragg spots measured in different crystal orientations (Clabbers & Abrahams, 2018 ▸; Subramanian *et al.*, 2015 ▸). The remaining dynamical scattering can be further corrected using the likelihood-based method described by Clabbers *et al.* (2019 ▸). As dynamical scattering increases the intensity of weak reflections and decreases the intensity of strong reflections, weak diffraction data are, on average, overestimated. Applying a statistical correction to reduce overestimated weak intensities as a function of resolution can therefore significantly improve structure-factor accuracy.

Another feature that distinguishes electron diffraction from other diffraction techniques is that electrons are scattered by the Coulomb potential in the crystal. Atomic scattering factors for electrons therefore depend strongly on the charge states of the atoms at low and medium resolution (Yonekura & Maki-Yonekura, 2016 ▸). This feature of electron diffraction offers a unique opportunity in structural biology as charged groups are central to protein functions such as enzyme catalysis (Warshel *et al.*, 2006 ▸). Metal ions, in particular, often act as catalytic or structural cofactors in enzymes that participate in important metabolic pathways. Identifying the partial charge states of metals in proteins will be crucial in understanding the reactivity of functional proteins. Refinement against electron diffraction data with different metal-ion charge states will allow us to detect their partial charges in protein crystals (Yonekura & Maki-Yonekura, 2016 ▸).

Here, we collected continuous rotation electron diffraction data from protein nanocrystals of bovine insulin, thermolysin from *Bacillus thermoproteolyticus* and thaumatin from *Thaumatococcus daniellii*. Thin crystals were chosen for data collection using a Timepix hybrid pixel detector. To further reduce the dynamical scattering component of the data, we applied the likelihood-based approach to correct for the systematic overestimation of weak electron diffraction intensities. Here, we improve the method of Clabbers *et al.* (2019 ▸) by including all measured intensities, including negative ones, in the calculation of correction parameters. We show that this correction notably improves crystallographic refinement. This improvement allowed an exploration of the scattering factors for various charge states of coordinated metal ions in insulin and thermolysin in the final refinement. For insulin, we also compared the electron diffraction results with X-ray diffraction results obtained from micrometre-sized crystals obtained from the same preparation.

## 2. Materials and methods   

### 2.1. Crystallization   

Insulin from bovine pancreas (Sigma, catalog No. I1882) was dissolved in double-distilled water to a concentration of 26 mg ml^−1^ in a 1.5 ml reagent tube. To improve the dissolution, the tube was gently agitated for approximately an hour at about 38°C until the solution remained clear. In a separate 1.5 ml reaction tube, the insulin solution was mixed with the crystallization solution (50 m*M* MES pH 6.5, 10 m*M* ZnCl_2_, 10 m*M* NaCl in double-distilled water) in a 1:4 ratio with a total volume of 250 µl. The tube was vortexed for 30 s directly after the two solutions had been added. Crystallization took place immediately in this batch setup and crystals in the size range 10 µm to ∼50 nm could be observed. Thaumatin and thermolysin crystals were a kind gift from Dr Ilme Schlichting and were from the same batch as used for XFEL diffraction experiments (Nass *et al.*, 2016 ▸).

### 2.2. Crystal handling and freezing   

Crystals of 10–50 µm in each dimension were diluted in distilled water (insulin and thaumatin) or buffer (thermolysin) and vortexed for a few minutes in an Eppendorf tube with a 3/32′′ PTFE bead (Smart Parts) for insulin and 2.381 mm PTFE MicroSeed beads (Molecular Dimensions, catalog No. MD2-14) for thaumatin and thermolysin. The vortexing time was adjusted in order to obtain a solution of crystals that contained a majority of sub-micrometre crystals suitable for electron diffraction.

The crystals were then applied onto electron-microscopy grids and vitrified. For the insulin sample, 2–3 µl crystal solution was deposited on the carbon-film side of Quantifoil or lacey carbon-coated copper grids. Extra solution was manually blotted away from the side opposite the sample-deposition side of the grid in order to keep the maximum number of crystals on the grid, and the grids were plunge-frozen in liquid ethane using an artisan plunge-freezer. Grids with crystals of thermolysin and thaumatin were prepared using a Vitrobot Mark IV (Thermo Fisher Scientific) with 100% humidity at 20°C. A volume of 2–3 µl of the crystal suspension was applied onto glow-discharged, lacey carbon-coated 300-mesh copper grids. After blotting for 3–4 s, the grids were plunge-frozen in liquid ethane cooled by liquid nitrogen.

### 2.3. Electron diffraction data collection and data processing   

The electron diffraction data were collected on Polara and Talos cryo-electron microscopes (Thermo Fisher) both operated at 200 keV and equipped with a Timepix hybrid pixel detector. Electron diffraction crystallographic data were collected from seven crystals of insulin (Supplementary Table S1), two crystals of thermolysin (Supplementary Table S2) and ten crystals of thaumatin (Supplementary Table S3). All data sets were processed with the *XDS* package (Kabsch, 2010 ▸). The structures of thermolysin and thaumatin were obtained by merging several diffraction data sets from cryocooled sub-micrometre-sized 3D crystals. Two data sets for the thermolysin structure and ten data sets for the thaumatin structure were merged. The two thermolysin crystals were continuously rotated from 17° through 34° with 0.186° per frame and 0.253° per frame in a 2 µm diameter beam. For thaumatin, all ten crystals were continuously rotated starting at 7° and ending at 14°. The data sets for thermolysin and thaumatin showed a decrease in the intensity of reflections with time and the last frames were thus discarded. The data were integrated with *XDS* and merged with *XSCALE* to 3.26 Å resolution for thermolysin and 2.76 Å resolution for thaumatin, resulting in an overall completeness of 84% and 66%, respectively (Table 1 ▸). For the insulin data sets, a significant decrease in the intensity of the highest resolution reflections was observed after about 60 s exposure time. Therefore, only the first 50–90 frames were used for data processing. Data sets 3 to 7 were merged with *XSCALE*, providing a rather complete 3.25 Å resolution set of unique reflections (Table 1 ▸). The statistics of data sets 1 and 2 indicated the presence of a merohedral twinning and were thus discarded.

To correct for the dynamical scattering, *Microsoft Excel* and *LibreOffice* were used to plot *I*_o_ versus |*F*_c_|^2^ and for least-squares fitting of the hyperbolic function with the observed and calculated amplitudes to derive the dynamical scattering error term *I*_e_. The scaling of the observed amplitudes and the experimental sigma was applied using the built-in functions and arithmetic operators available in *SFTOOLS* (Winn *et al.*, 2011 ▸), as described in the supporting information.

### 2.4. X-ray data collection and data processing   

A 50 µm crystal was taken from the crystal preparation used in electron diffraction experiments, soaked for about 30 s in a cryoprotectant solution containing 25% glycerol and flash-cooled in liquid nitrogen. Crystallographic data were collected at 110 K on the BM30A beamline of the European Synchrotron Research Facility (ESRF) using an ADSC Quantum 315r CCD detector. Data were processed with the *XDS* package (Kabsch, 2010 ▸). A complete 2.3 Å resolution data set was obtained, as shown in Table 1 ▸.

### 2.5. Parametrization of electron atomic scattering factors for partially charged ions   

Electron scattering factors for zinc and Zn^2+^ were taken from Volume C of *International Tables for Crystallography* (Prince, 2006 ▸) and were parametrized using a five-Gaussian model as required by *REFMAC* (the fit to determine the five Gaussian parameters was performed with *Fityk* in the range 25–1 Å). Electron scattering factors for intermediate charges were approximated by a linear combination of zinc and Zn^2+^ atomic scattering factors (Yonekura & Maki-Yonekura, 2016 ▸) and parametrized by the same method (Supplementary Table S4). The library of electron atomic scattering factors provided by CCP4 (atomsf_electron.lib) was complemented with the different charge states of Zn and used for refinement in *REFMAC*.

### 2.6. Structure refinement   

The structures were solved by molecular replacement with *Phaser* from the *CCP*4 suite of programs (Winn *et al.*, 2011 ▸) using the structure of bovine insulin from PDB entry 2a3g (Smith *et al.*, 2005 ▸), the structure of *T. daniellii* thaumatin from PDB entry 6c5y (Guo *et al.*, 2018 ▸) and the structure of *B. thermoproteolyticus* thermolysin from PDB entry 1fj3 (English *et al.*, 2001 ▸) as search models. The final structures were obtained by several rounds of manual building with the *Coot* software (Emsley *et al.*, 2010 ▸) and maximum-likelihood refinement with *REFMAC*5 from the *CCP*4 suite. For refinement against electron diffraction data, the electron atomic form factors provided by the CCP4 library (atomsf_electron.lib) were used by specifying source EC in the* REFMAC*5 script. The CCP4 library was complemented with the different charge states of Zn, Ca and O and used for refinement in *REFMAC*. A likelihood-based correction was performed to reduce the overestimation of lower intensities by dynamical scattering (Clabbers *et al.*, 2019 ▸). The refinement statistics are detailed in Table 1 ▸.

## 3. Results   

### 3.1. Overall description of the structures   

The data-processing and refinement statistics for the three proteins are shown in Table 1 ▸. The structure of bovine insulin was determined by both X-ray and electron diffraction using crystals from the same batch, *i.e.* grown under the exact same conditions. The X-ray structure was refined at 2.3 Å resolution, which was sufficient to yield a reference structure for the electron diffraction structure. The structure is very similar to the structure of T_6_ bovine insulin (PDB entry 2a3g; r.m.s.d. of 0.34 Å on C^α^ atoms) and belongs to the same space group with similar unit-cell parameters, despite being obtained from crystals grown under different crystallization conditions. The asymmetric unit contains two insulin molecules as well as two zinc ions and two chloride ions located on the crystallographic threefold axis and 23 water molecules. The electron density of these insulin crystals is well defined for all amino acids except for residue 30 of chain B and residues 1 and 2 of chain D.

The insulin structure refined against electron diffraction data at 3.25 Å resolution agrees well with the X-ray structure refined at 2.3 Å resolution in this study (r.m.s.d. of 0.38 Å on C^α^ atoms). Similar to the electron-density map from X-ray diffraction, the Coulomb potential map is well defined along all of the polypeptide chains, except for residues 29–30 of chain B and residues 1 and 2 of chain D. However, no solvent molecules could be identified in the Coulomb potential map except for one zinc ion, which is clearly visible in the map and corresponds to that chelated by His10 of chain D (and its two symmetry mates). The zinc site in the B chain is vacant. This observation is consistent with the fact that the insulin crystals were diluted and crushed in pure water, which might have caused zinc ions to diffuse out of the nanocrystals during the vortexing step (see Section 2). Moreover, the overall *B* factor estimated from the Wilson plot is higher for the electron diffraction data (71.9 Å^2^) than for the X-ray diffraction data (35.6 Å^2^), suggesting that the crushing step and/or the loss of zinc ions may have introduced some disorder into the insulin crystals.

The thermolysin structure refined against electron diffraction data at 3.26 Å resolution also exhibits a very good fit with the Coulomb potential map for all 316 residues. One zinc ion and four calcium ions could clearly be observed in the map and were included in the refined model. The final model is very similar to the X-ray crystallographic structure of the same protein at 2.0 Å resolution (PDB entry 1fj3), with an r.m.s.d. of 0.49 Å for 314 C^α^ atom pairs.

The thaumatin structure was refined against electron diffraction data to 2.76 Å resolution. The fit with the Coulomb potential map is good for all 207 residues. The structure is similar to PDB entry 6c5y, an X-ray crystallographic structure refined at 2.5 Å resolution obtained from microcrystals (r.m.s.d. of 0.42 Å for 202 C^α^ atom pairs). No solvent molecules were identified except for one putative chloride ion on a twofold axis coordinated by the guanidinium group of Arg82.

Whereas the refinement statistics given in Table 1 ▸ are far from ideal compared with X-ray crystallographic standards, they are in line with previous structures refined against electron diffraction data at similar resolution. Indeed, previous protein structures solved by 3D electron diffraction deposited in the PDB in the 2.7–3.4 Å resolution range exhibit *R*_work_ and *R*_free_ values in the ranges 0.21–0.32 and 0.25–0.34, respectively. The gap between *R*_work_ and *R*_free_ increases as expected when the data-to-parameter ratio decreases, as observed in the different data sets here. In general, refinement against X-ray diffraction data yields better values for *R*_work_ than refinement against electron diffraction data. Undoubtedly, multiple scattering and lower *I*/σ(*I*) ratios contributed to this discrepancy. The relatively large gap observed for the electron diffraction data of insulin is likely to be owing to both the low resolution (3.25 Å) and the low solvent content (∼35%) in the crystals. The rather low overall completeness of the thaumatin data comes from the fact that only one crystal diffracted to 2.76 Å resolution, while the others diffracted to 3.10–3.77 Å resolution (see Supplementary Table S3). As a consequence, the data are 85.6% complete at 3.19 Å resolution and 81.9% complete at 3.09 Å resolution, but only 22.6% complete in the 3.09–2.76 Å resolution shell. Although all available reflections were included in refinement in order to obtain the most accurate model, the actual resolution of the final thaumatin model is closer to 3.1 Å.

### 3.2. Applying the dynamical scattering correction   

We previously observed an overestimation of the weaker structure-factor amplitudes |*F*_o_(**h**)| compared with calculated amplitudes from the structural model |*F*_c_(**h**)| in the electron diffraction of small molecules (Clabbers *et al.*, 2019 ▸). We attributed this effect to a dynamical electron scattering term |*F*_e_| and described this trend using a hyperbolic function defining the expected value of |*F*_o_(**h**)| as
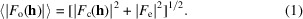



Using this relation, we derived the expected dynamical scattering error term |*F*_e_| using least-squares fitting. The dynamical error term |*F*_e_| was used to determine a scaling factor *k*(**h**) to be applied to both the observed amplitude |*F*_o_(**h**)| and experimental sigmas,
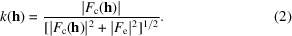



This method has yielded good results on strongly diffracting crystals of small molecules and a relatively strong lysozyme data set. This approach, however, cannot be applied directly to correct data from weakly diffracting protein crystals such as those in this study.

Electron diffraction data from protein crystals are inherently weak owing to the limited electron dose that each crystal can tolerate without noticeable damage. A significant fraction of weak intensities is measured as being negative (in our data sets 12% for thermolysin, 10% for thaumatin and 6% for insulin) owing to counting statistics (Hattne *et al.*, 2016 ▸). For these very weak intensities, calculation of the structure factor is not straightforward and the *TRUNCATE* procedure implemented in *CCP*4 (Winn *et al.*, 2011 ▸) provides a better estimation of the structure factor than just taking the square root of the intensity for positive values and zero for negative values (French & Wilson, 1978 ▸). Refinement tests on the thermolysin electron diffraction data confirmed that using the truncated structure factors allows more reflections to be used in the refinement, leading to a slightly lower gap between the *R*_work_ and *R*_free_ values and better stereochemistry. However, using these truncated structure factors for dynamical scattering data correction led to an unrealistic improvement of the refinement statistics, especially in the high-resolution shell, where truncated structure factors derived from negative intensities are most abundant.

In order to avoid any possible bias in the correction introduced by these reflections, we adapted the likelihood-based correction described in Clabbers *et al.* (2019 ▸) to calculate the correction parameters by comparing *I*_o_(**h**) against |*F*_c_(**h**)|^2^ instead of |*F*_o_(**h**)| against |*F*_c_(**h**)| (see the supporting information for more detailed information). The scaling factor *k*(**h**) used for the intensity-based corrections with the dynamical error term *I*_e_ is now described as
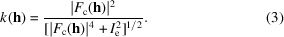



**Figure 1:**
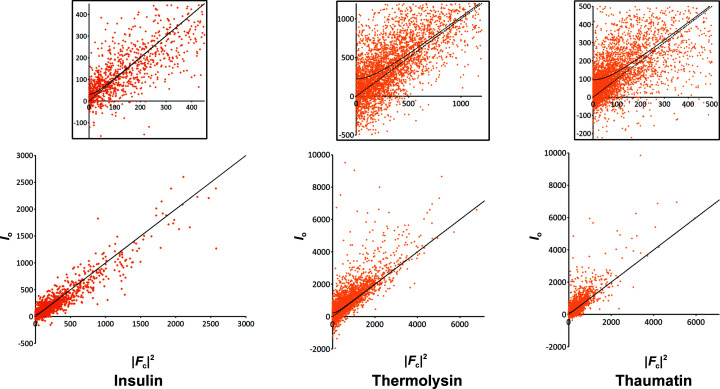
Intensity *I*_0_ versus |*F*_c_|^2^ plots for insulin, thermolysin and thaumatin. The solid line shows the best least-squares fit of a hyperbolic function to the observed intensity *I*_0_ as described in the supporting information. The dotted line shows the case when *I*_0_ relates simply to |*F*_c_|^2^. The top panels enlarge the regions where the solid lines deviate from the dotted lines. The 〈*I*_o_〉 and *I*_e_ values are 283.9 and 31.6, respectively, for insulin, 753.0 and 226.3, respectively, for thermolysin, and 294.0 and 96.1, respectively, for thaumatin.

**Figure 2:**
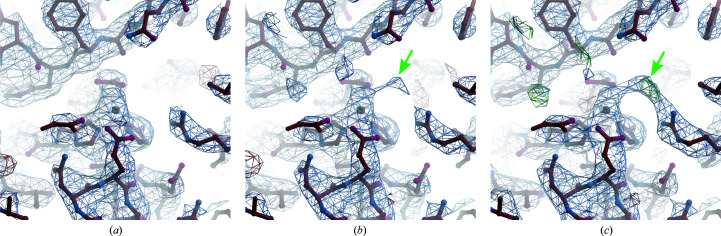
Calculated Coulomb potential map of thermolysin with the atomic model in this study showing the active site with the zinc ion. The structure is determined from data merged from two nanocrystals. The (2*F*_obs_ − *F*_calc_) map is contoured at the 1σ level and is depicted in blue. The residual (*F*_obs_ − *F*_calc_) map is contoured at the +3σ level (depicted in green) and the −3σ level (depicted in red). (*a*) Before correcting for dynamical scattering. (*b*) After correcting for dynamical scattering. (*c*) After correcting for dynamical scattering and considering the zinc charge. The positive peak in the residual map that appears in the final model (green arrow) could be attributed to a hydroxy or chloride ion, which bridges the zinc ion and the guanidinium group capping Arg203.

Such a procedure allows negative intensities to be included in the likelihood-based correction and yields a more realistic estimation of dynamical scattering on weak reflections (Fig. 1 ▸). The corrections were calculated with the final model refined against uncorrected data and the corrected data were then used to run 30 successive *REFMAC* runs of 20 cycles each to ensure that both *R*_work_ and *R*_free_ reached convergence. Table 1 ▸ shows that the correction always improves both *R*_work_ and *R*_free_, while the stereochemistry remains essentially identical. The gap between *R*_work_ and *R*_free_ is also slightly smaller, except for the thaumatin data. Moreover, the noise level has decreased in the difference Coulomb potential maps. For thermolysin, the corrected map revealed the presence of a solvent molecule that could be either a water molecule, a hydroxy ion or a chloride ion bridging the guanidinium group of Arg203 and the zinc ion (Fig. 2 ▸). For comparison, in PDB entry 1fj3 an acetone molecule is observed at this location, with the acetone O atom forming a similar bridge between the guanidinium group and the zinc ion.

### 3.3. Charge analysis of bound ions   

Since the electron atomic scattering factor depends strongly on the charge of the atom, we next evaluated the possibility of using the electron diffraction data collected in this study to analyze the charge states of the metal ions present in insulin (zinc) and thermolysin (zinc and calcium). As electrons interact with matter in a different manner to X-rays, the photoreduction of metal centers is not a concern in electron diffraction experiments (Kekilli *et al.*, 2017 ▸). Whereas an inelastic X-ray photon loses all of its energy owing to the photoelectric effect, an inelastic electron deposits only a fraction of its energy through plasmon excitation, which upon relaxation releases far less energy (in the 10–100 eV range) than X-rays (Henderson, 1995 ▸). [Radiolysis, radiation damage from an electron beam that could alter charge states, has a small cross section (Egerton, 2012 ▸).] Electron diffraction would therefore be more appropriate for charge analysis than X-ray diffraction.

The formal charges of both zinc and calcium ions are +2 in the crystallization solution, as the zinc and calcium ions originate from the dilution of ZnCl_2_ and CaCl_2_ salts, respectively. However, according to *ab initio* calculations or electronegativity equalization methods, the actual charge of Zn atoms in protein molecules is often lower and may vary between +0.5 and +1.1 depending on the zinc ligands and the calculation method (Abdallah *et al.*, 2009 ▸; Shen *et al.*, 1990 ▸). For instance, the zinc ion present in αB-crystallin, which is bound to three histidines and a glutamic acid that adopt a tetrahedral geometry as in insulin, has been estimated to have a partial charge of +0.72 by electrostatic surface potential calculations (Coi *et al.*, 2005 ▸).

In our insulin X-ray structure, a zinc ion was observed in both chains (B and D) at full occupancy. This zinc ion (and its symmetry mates) lies on the crystallographic threefold axis and is coordinated by His10 NE2 (Zn–His10 NE2 distance of 2.05 Å). The zinc *B* factor (28.8 and 27.8 Å^2^) is similar to that of His10 NE2 (27.8 and 28.5 Å^2^). The similarity of the *B* factors of zinc and His10 suggests strong binding of the zinc ion. In our refined electron diffraction structure, only one Zn atom of the insulin could be modeled. This Zn atom is chelated to His10D with a similar coordination distance (1.96 Å). When a full occupancy neutral zinc atomic scattering factor is used, the atom has a *B* factor of 19.5 Å^2^, which remains essentially the same after refinement against data corrected for multiple scattering (19.6 Å^2^). As observed in Table 2 ▸, this zinc *B* factor is surprisingly lower than the *B* factor of the chelating His10D (31 Å^2^). The discrepancy suggests that the use of a positively charged zinc atomic scattering factor is more appropriate for this position.

**Figure 3:**
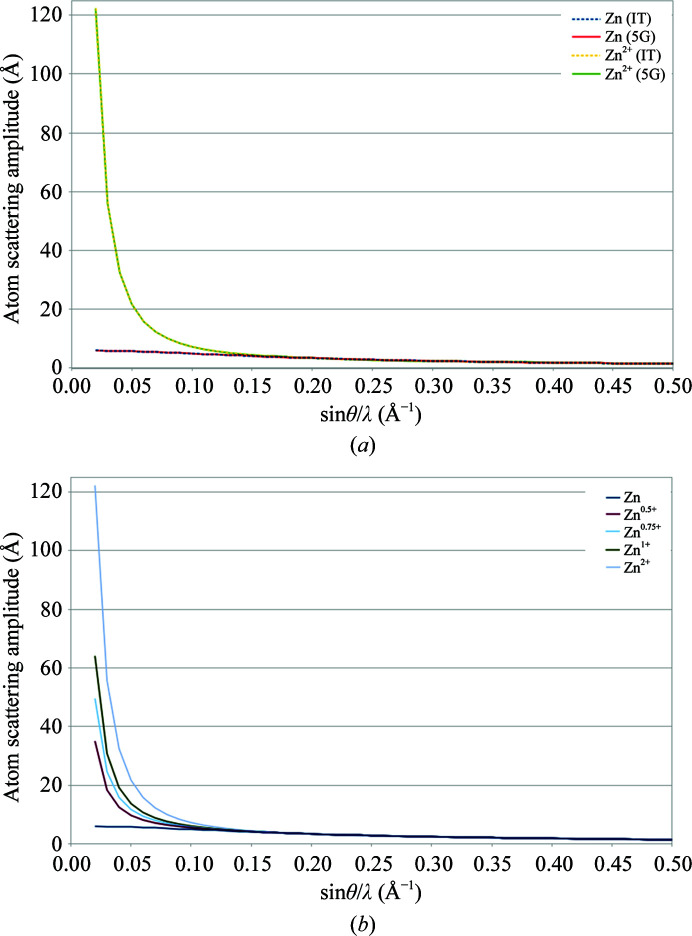
Electron scattering factors for charged Zn atoms. (*a*) The five-Gaussian (5G) parametrizations for neutral and +2 charged Zn atoms follow the red and green solid lines, respectively. The electron atomic scattering curves derived from *International Tables for Crystallography* Volume C (IT) for zinc and Zn^2+^ are shown by the blue and yellow dashed lines. The quasi-perfect fit illustrates the accuracy of the five-Gaussian parametrization in the 25–1 Å resolution range and the very low *R*_scat_ values (see Supplementary Table S4). (*b*) Atomic scattering factors for various charge states of zinc, as calculated from the linear combination of neutral and +2 charge states.

**Figure 4:**
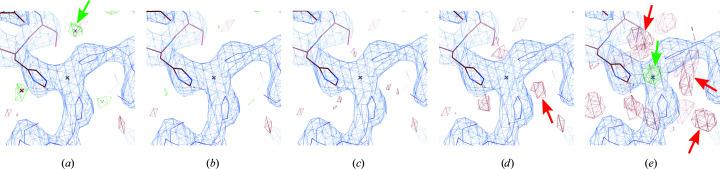
The atomic model and the calculated Coulomb potential map of insulin at one of the zinc ion sites for different assigned zinc charge states: (*a*) neutral, (*b*) +0.5, (*c*) +0.75, (*d*) +1.0, (*e*) +2.0. The (2*F*_obs_ − *F*_calc_) map is contoured at the 1σ level and is depicted in blue. The residual (*F*_obs_ − *F*_calc_) map is contoured at the +3σ level (depicted in green) and −3σ level (depicted in red). The zinc ion is coordinated by three histidines. Green and red arrows point to residual Coulomb potential map peaks that appeared when improper charge states are used. The +0.75 charge yields the optimal residual map.

We analyzed our data following the approach employed by Yonekura and Maki-Yonekura to analyze electron diffraction data from catalase and β-galactosidase crystals (Yonekura & Maki-Yonekura, 2016 ▸). We first calculated the electron atomic scattering factors for different charge states of zinc and calcium ions (+0.5, +0.75, +1.0 and +2.0) in the resolution range 25–1 Å and proceeded to a five-Gaussian parametrization in order to use these atomic scattering factors in *REFMAC* (Supplementary Table S4). We obtained *R*_scat_ values, as defined by Yonekura & Maki-Yonekura (2016 ▸), below 0.23%, indicating that the five-Gaussian parametrization provided an accurate model of the electron atomic scattering factors of zinc and calcium ions (Fig. 3 ▸ *a*). The last refinement step with *REFMAC* was then performed using the different zinc atomic scattering-factor parameters. The *B* factors and refinement statistics are shown in Table 2 ▸. Given that the zinc ion should have a similar *B* factor as its chelating histidine (as is the case in the X-ray insulin structure), we estimated the zinc ion charge to be between +0.75 and +1. This estimate is supported by the inspection of the Coulomb potential map for the different charge states, as shown in Fig. 4 ▸, which shows a good agreement between the map and the model for the case of a zinc charge of +0.75.

The same procedure was applied to the thermolysin structure, in which one zinc ion and four calcium ions were identified in the Coulomb potential map. Initially, different zinc charge states were tested (0, +0.5, +0.75, +1.0 and +2.0). Unexpectedly, the zinc *B* factor obtained with uncorrected data did not smoothly increase with the charge, as would be expected given the increase in the atomic electron scattering factor with the charge value (Fig. 3 ▸ *b*). As shown in Table 3 ▸, the zinc *B* factor for the uncorrected data is relatively stable for charges between 0 and +1 (with even a slight decrease at +1) and increases for charge +2. In contrast, the zinc *B* factor obtained with data corrected for dynamical scattering regularly increases with the charge, demonstrating that the data correction has improved the agreement with Wilson statistics and has resulted in more realistic *B* factors. Comparing the *B* factor of zinc and that of the chelating atoms from data corrected for dynamical scattering (Table 3 ▸) shows that the actual zinc charge is close to +0.75 for thermolysin, as in the case of insulin.

The charge of the four calcium ions in thermolysin was investigated using the same approach and the results are shown in Table 4 ▸. The charge of the zinc ion was set to +2 for the uncorrected data and +0.75 for the corrected data because these charge values yielded the most realistic zinc *B* factors compared with the *B* factors of its ligands. Similar to the case of the zinc ion, the uncorrected data led to an unexpected evolution of the *B* factors, whereas the corrected data exhibited a smooth increase in calcium *B* factors with the charge value. Examining the entries in Table 4 ▸ suggests that the actual charge of the calcium ions is between +0.75 and +1. The actual value is likely to be closer to +1, where the calcium *B* factor is higher or comparable to the *B* factors of all of the chelating residues. It is interesting to note that the *R*_work_ and *R*_free_ values are not much affected by the charge of either the zinc or the calcium ions for charges in the 0 to +1 range. Therefore, these indicators cannot be reliably used to estimate the actual atomic charge. However, *R*_work_ and *R*_free_ are systematically significantly higher when the +2 formal charge is used, further supporting our analysis based on the *B* factor.

We also noticed that the Coulomb potential map is rather weak around the various carboxylic groups involved in the coordination of the zinc or calcium ions. Accordingly, we tested applying a negative charge to the O atom of these carboxylic groups. Electron atomic scattering factors for this partially charged O atom were taken from Yonekura & Maki-Yonekura (2016 ▸). Charges from −0.1 to −0.5 were tested and a charge of −0.3 resulted in the lowest *R* factor.

## 4. Discussion   

Our results confirm that a statistical correction for the overestimation of the intensity of weak reflections arising from dynamical scattering can be generally extended to protein crystals. The correction procedure was originally developed for organic molecule electron diffraction data and to date had only been demonstrated for protein data using a single lysozyme test case. Here, it has been tested on three different protein crystals, and in all of the cases it improved both the refinement statistics and the Coulomb potential map when the corrections are calculated from intensities instead of structure factors, a procedure that allows negative intensities from weak reflections to be included.

Moreover, the correction of the data for dynamical scattering significantly improves the stability of *B*-factor refinement of ions, which allowed us to estimate the charges of ions in this study. We were able to analyze the charge of several metal ions in insulin and thermolysin crystals, as has been performed for other protein–metal complexes (Yonekura *et al.*, 2015 ▸). The zinc ion charge derived from our electron diffraction data agrees well with the partial charge estimated by the electrostatic surface potential calculation for a zinc ion in a similar binding site (Coi *et al.*, 2005 ▸). Calcium ions in thermolysin are estimated to have a partial charge of close to +1.

At first sight, the quality of our electron diffraction data seems inferior compared with X-ray diffraction data even after corrections, in terms of both resolution and the *I*/σ(*I*) ratio. The thermolysin and thaumatin crystals originate from the same batch of nanocrystals previously analyzed by serial crystallography using an X-ray free-electron laser (XFEL) source, which yielded structures with a resolution of 2.1 Å for both thermolysin (Hattne *et al.*, 2014 ▸) and thaumatin (Nass *et al.*, 2016 ▸). However, after the XFEL experiments, the protein had recrystallized into larger crystals with a size range of 10–50 µm, which is orders of magnitude too large for electron diffraction experiments. We therefore had to crush these crystals by vortexing the crystals with Teflon beads (see Section 2). We cannot exclude the possibility that this procedure compromised the internal ordering of the crystals.

Nonetheless, we believe that the inferior quality of the present electron diffraction data can be largely explained by the difference in the number of unit cells contributing to the signals in the two types of experiments. The electron diffraction results came from only a few thin crystals. These crystals had dimensions in the sub-micrometre range, with a maximal thickness of up to ∼200 nm. In comparison, about 11 600 and 125 000 crystals of average sizes 2 × 3 × 1 and 3 × 3 × 5 µm contributed to the XFEL diffraction data of thermolysin and thaumatin, respectively. Given the size of the XFEL X-ray beam (2.25 µm^2^), the diffracting volumes per crystal are in the 2–7 and 7–11 µm^3^ ranges for thermolysin and thaumatin, respectively. There is thus a difference of around six to seven orders of magnitude in the crystal volumes used in the two types of experiments. This difference may fully account for the difference in resolution limit. Similar differences in crystal volumes can also explain the higher quality X-ray synchrotron data for the insulin crystals over the electron diffraction data.

In order to fill this gap in data quality, serial electron crystallography is developing quickly and yields a substantial improvement in structure quality (Smeets *et al.*, 2018 ▸). Very recently, serial electron diffraction data obtained from naturally produced granulovirus polyhedrin crystals (Bücker *et al.*, 2020 ▸) led to higher resolution data than a serial crystallo­graphy XFEL experiment using crystals of comparable size (Gati *et al.*, 2017 ▸), affirming the quality of electron diffraction data when the number of contributing unit cells is comparable. A semi-automatic protocol has also been developed for electron diffraction data collection with continuous rotation, which can significantly improve the throughput of data production (de la Cruz *et al.*, 2019 ▸; Takaba *et al.*, 2020 ▸; Wang *et al.*, 2019 ▸). Such results and the results presented here experimentally confirm the added value of electron diffraction as a structural biology technique, especially in cases when only ;sub-micrometre-sized crystals are available and when the charge states of component atoms or groups are of interest.

## Supplementary Material

PDB reference: insulin, electron diffraction data, 6zhb

PDB reference: X-ray diffraction data, 6zi8

PDB reference: thermolysin, electron diffraction data, 6zhj

PDB reference: thaumatin, electron diffraction data, 6zhn

Supplementary Tables, Supplementary Figure and data correction for dynamical scattering. DOI: 10.1107/S2059798320014540/di5040sup1.pdf

## Figures and Tables

**Table:** (*a*) Data processing.

	Insulin X-ray data	Insulin ED data (data sets 3–7 merged)	Thermolysin	Thaumatin
Space group	*R*3	*R*3	*P*6_1_22	*P*4_1_2_1_2
Unit-cell parameters (Å)	*a* = *b* = 81.28, *c* = 33.31	*a* = *b* = 82.40, *c* = 33.46	*a* = *b* = 92.00, *c* = 127.48	*a* = *b* = 57.72, *c* = 149.17
Wavelength (Å)	0.979855	0.02508	0.02508	0.02508
Resolution range (Å)	30.73–2.30 (2.36–2.30)	30.73–3.25 (3.40–3.25)	14.58–3.26 (3.34–3.26)	12.34–2.76 (2.83–2.76)
No. of observations	16882 (660)	3194 (398)	18806 (679)	21293 (243)
No. of unique reflections	3613 (239)	1201 (131)	4536 (282)	4597 (112)
Multiplicity	4.7 (2.8)	2.7 (3.0)	4.2 (2.4)	4.6 (2.2)
Completeness (%)	99.3 (93.7)	90.2 (85.6)	84.3 (71.6)	65.6 (23.1)†
*R*_merge_	0.095 (0.282)	0.272 (0.747)	0.548 (0.781)	0.537 (0.803)
*R*_meas_	0.107 (0.347)	0.328 (0.891)	0.618 (1.001)	0.593 (1.055)
CC_1/2_	0.993 (0.880)	0.923 (0.496)	0.867 (0.455)	0.878 (0.410)
〈*I*/σ(*I*)〉	11.81 (3.78)	2.86 (1.29)	2.14 (1.02)	2.09 (1.00)
Wilson *B* factor (Å^2^)	35.6	71.9	40.3	37.3
Twin fraction (*SFCHECK*, *H*-test)	0.036	0.072	0.073	0.039

**Table:** (*b*) Refinement.

		Insulin ED data (data sets 3–7 merged)		Thermolysin		Thaumatin	
	Insulin X-ray data	Uncorrected data	Corrected for dynamical scattering	Uncorrected data	Corrected for dynamical scattering	Uncorrected data	Corrected for dynamical scattering
Resolution range (Å)	24.20–2.30 (2.36–2.30)	30.30–3.25 (3.33–3.25)	30.30–3.25 (3.33–3.25)	37.52–3.26 (3.34–3.26)	37.49–3.26 (3.34–3.26)	12.34–2.76 (2.83–2.76)	12.34–2.76 (2.83–2.76)
No. of reflections							
Work set	3213 (217)	1019 (64)	1030 (63)	4293 (262)	4293 (262)	4364 (108)	4364 (108)
Free set	180 (9)	108 (4)	97 (5)	237 (12)	237 (12)	231 (3)	231 (3)
No. of atoms	805	770	770	2438	2438	1551	1551
No. of reflections/No. of parameters	0.998	0.334	0.334	0.440	0.440	0.703	0.703
*R* factor	0.166	0.194	0.187	0.214	0.187	0.283	0.255
*R*_work_	0.162 (0.130)	0.181 (0.349)	0.178 (0.296)	0.210 (0.347)	0.184 (0.213)	0.281 (0.160)	0.253 (0.323)
*R*_free_	0.238 (0.333)	0.319 (0.501)	0.272 (0.338)	0.292 (0.541)	0.238 (0.416)	0.320 (0.176)	0.295 (0.276)
σ, bonds (Å)	0.008	0.006	0.006	0.007	0.006	0.005	0.005
σ, angles (°)	1.174	1.034	1.011	1.156	1.057	1.005	1.008

† Only one crystal diffracted to 2.76 Å resolution (see Supplementary Table S3 for details). The completeness of the thaumatin data is 85.6% at 3.19 Å resolution, 81.9% at 3.09 Å resolution and 22.6% in the 3.09–2.76 Å resolution shell.

**Table 2:** Comparison of refinement statistics for insulin Uncorrected data and data corrected for dynamical scattering, as well as different charge states of the Zn atom, are compared.

	Zn		Zn^0.5+^		Zn^0.75+^		Zn^1+^		Zn^2+^	
	Uncorrected data	Corrected data	Uncorrected data	Corrected data	Uncorrected data	Corrected data	Uncorrected data	Corrected data	Uncorrected data	Corrected data
Occupancy	1	1	1	1	1	1	1	1	1	1
Zinc *B* factor (Å^2^)	19.39	19.49	23.31	23.96	23.65	27.83	39.36	34.33	82.41	418.6
〈*B* factor〉 (His10D NE2) (Å^2^)	31.07	33.48	32.32	34.41	32.70	34.58	32.95	34.85	29.95	38.79
*R*_work_	0.181	0.177	0.185	0.184	0.184	0.184	0.188	0.186	0.196	0.193
*R*_free_	0.319	0.298	0.313	0.275	0.327	0.278	0.308	0.279	0.318	0.283

**Table 3:** Comparison of refinement statistics for thermolysin Uncorrected data and data corrected for dynamical scattering, as well as different charge states of the Zn atom, are compared.

	Zn		Zn^0.5+^		Zn^0.75+^		Zn^1+^		Zn^2+^	
	Uncorrected data	Corrected data	Uncorrected data	Corrected data	Uncorrected data	Corrected data	Uncorrected data	Corrected data	Uncorrected data	Corrected data
Zinc *B* factor (Å^2^)	16.34	24.25	16.07	28.23	17.47	35.78	15.35	43.38	37.11	84.27
〈*B* factor〉 (His142 NE2, His146 NE2, Glu143 OE2, Glu166 OE2, Tyr157 OH, His231 NE2) (Å^2^)	28.51	33.92	28.81	32.50	29.10	33.01	28.74	33.59	28.51	32.33
*R*_work_	0.210	0.184	0.211	0.175	0.211	0.175	0.211	0.175	0.202	0.179
*R*_free_	0.292	0.238	0.292	0.240	0.292	0.240	0.298	0.240	0.298	0.251

**Table 4:** Comparison of refinement statistics for thermolysin Uncorrected data and data corrected for dynamical scattering, as well as different charge states of the Ca atoms, are compared. The charge of Zn was set to +2 for the uncorrected data and to +0.75 for the corrected data, as they provided the most realistic *B*-factor value for the Zn atom.

	Ca		Ca^0.5+^		Ca^0.75+^		Ca^1+^		Ca^2+^	
	Uncorrecteddata	Corrected data	Uncorrected data	Corrected data	Uncorrecteddata	Corrected data	Uncorrected data	Corrected data	Uncorrected data	Corrected data
Ca1 *B* factor (Å^2^)	18.21	28.83	15.08	33.47	14.87	36.22	16.29	38.29	12.09	61.91
〈*B* factor〉 (Asp138 OD2, Glu177 OE1, Glu177 OE2, Asp185 OD1, Glu187 O, Glu190 OE1, Glu190 OE2) (Å^2^)	35.09	38.39	34.58	40.36	33.53	41.59	32.68	38.42	28.02	36.57
Ca2 *B* factor (Å^2^)	29.56	38.61	34.39	43.84	34.92	46.92	30.61	56.51	62.45	103.78
〈*B* factor〉 (Glu177 OE2, Asn183 O, Asp185 OD1, Glu190 OE2) (Å^2^)	36.58	39.84	36.77	41.61	36.20	42.69	34.93	40.43	32.32	39.55
Ca3 *B* factor (Å^2^)	18.51	26.38	21.98	26.01	18.27	26.73	11.63	30.88	28.80	52.69
〈*B* factor〉 (Asp57 OD1, Asp57 OD2, Asp59 OD1, Gln61 O) (Å^2^)	25.70	30.86	24.18	30.85	22.28	31.64	21.17	28.54	17.98	27.40
Ca4 *B* factor (Å^2^)	31.50	28.46	39.51	32.15	34.62	34.06	24.33	37.59	46.14	58.75
〈*B* factor〉 (Glu190 O, Tyr193 O, Thr194 O, Thr194 OG1, Ile197 O, Asp200 OD1) (Å^2^)	39.42	43.74	37.09	44.56	35.72	45.34	35.47	41.85	28.89	41.56
*R*_work_	0.202	0.175	0.200	0.179	0.201	0.183	0.205	0.184	0.212	0.201
*R*_free_	0.298	0.240	0.309	0.243	0.303	0.246	0.298	0.251	0.313	0.268

## References

[bb1] Abdallah, H. H., Gadzhiev, O. B. & Adnan, R. (2009). *Proceedings of the 13th International Electronic Conference on Synthetic Organic Chemistry*, edited by J. A. Seijas & M. P. Vázquez Tato, https://doi.org/10.3390/ecsoc-13-00245. Basel: MDPI.

[bb2] Beale, E. V., Waterman, D. G., Hecksel, C., van Rooyen, J., Gilchrist, J. B., Parkhurst, J. M., de Haas, F., Buijsse, B., Evans, G. & Zhang, P. (2020). *Front. Mol. Biosci.* **7**, 179. 10.3389/fmolb.2020.00179 PMC7417479 32850967

[bb3] Bücker, R., Hogan-Lamarre, P., Mehrabi, P., Schulz, E. C., Bultema, L. A., Gevorkov, Y., Brehm, W., Yefanov, O., Oberthür, D., Kassier, G. H. & Miller, R. J. D. (2020). *Nat. Commun.* **11**, 996. 10.1038/s41467-020-14793-0 PMC7035385 32081905

[bb4] Clabbers, M. T. B. & Abrahams, J. P. (2018). *Crystallogr. Rev.* **24**, 176–204.

[bb5] Clabbers, M. T. B., Gruene, T., Parkhurst, J. M., Abrahams, J. P. & Waterman, D. G. (2018). *Acta Cryst.* D**74**, 506–518. 10.1107/S2059798318007726 PMC6096487 29872002

[bb7] Clabbers, M. T. B., Gruene, T., van Genderen, E. & Abrahams, J. P. (2019). *Acta Cryst.* A**75**, 82–93. 10.1107/S2053273318013918 PMC6302931 30575586

[bb8] Clabbers, M. T. B., van Genderen, E., Wan, W., Wiegers, E. L., Gruene, T. & Abrahams, J. P. (2017). *Acta Cryst.* D**73**, 738–748. 10.1107/S2059798317010348 PMC5586247 28876237

[bb9] Coi, A., Bianucci, A. M., Ganadu, M. L. & Mura, G. M. (2005). *Int. J. Biol. Macromol.* **36**, 208–214. 10.1016/j.ijbiomac.2005.06.009 16098576

[bb90] Cruz, M. J. de la, Hattne, J., Shi, D., Seidler, P., Rodriguez, J., Reyes, F. E., Sawaya, M. R., Cascio, D., Weiss, S. C., Kim, S. K., Hinck, C. S., Hinck, A. P., Calero, G., Eisenberg, D. & Gonen, T. (2017). *Nat. Methods*, **14**, 399–402. 10.1038/nmeth.4178 PMC5376236 28192420

[bb10] Cruz, M. J. de la, Martynowycz, M. W., Hattne, J. & Gonen, T. (2019). *Ultramicroscopy*, **201**, 77–80. 10.1016/j.ultramic.2019.03.009 PMC6752703 30986656

[bb11] Duyvesteyn, H. M. E., Kotecha, A., Ginn, H. M., Hecksel, C. W., Beale, E. V., de Haas, F., Evans, G., Zhang, P., Chiu, W. & Stuart, D. I. (2018). *Proc. Natl Acad. Sci. USA*, **115**, 9569–9573. 10.1073/pnas.1809978115 PMC6156647 30171169

[bb12] Egerton, R. F. (2012). *Microsc. Res. Tech.* **75**, 1550–1556. 10.1002/jemt.22099 22807142

[bb13] Emsley, P., Lohkamp, B., Scott, W. G. & Cowtan, K. (2010). *Acta Cryst.* D**66**, 486–501. 10.1107/S0907444910007493 PMC2852313 20383002

[bb96] English, A. C., Groom, C. R. & Hubbard, R. E. (2001). *Protein Eng.* **14**, 47–59. 10.1093/protein/14.1.47 11287678

[bb14] French, S. & Wilson, K. (1978). *Acta Cryst.* A**34**, 517–525.

[bb15] Gati, C., Oberthuer, D., Yefanov, O., Bunker, R. D., Stellato, F., Chiu, E., Yeh, S.-M., Aquila, A., Basu, S., Bean, R., Beyerlein, K. R., Botha, S., Boutet, S., DePonte, D. P., Doak, R. B., Fromme, R., Galli, L., Grotjohann, I., James, D. R., Kupitz, C., Lomb, L., Messerschmidt, M., Nass, K., Rendek, K., Shoeman, R. L., Wang, D., Weierstall, U., White, T. A., Williams, G. J., Zatsepin, N. A., Fromme, P., Spence, J. C. H., Goldie, K. N., Jehle, J. A., Metcalf, P., Barty, A. & Chapman, H. N. (2017). *Proc. Natl Acad. Sci. USA*, **114**, 2247–2252. 10.1073/pnas.1609243114 PMC5338516 28202732

[bb16] Gemmi, M., Mugnaioli, E., Gorelik, T. E., Kolb, U., Palatinus, L., Boullay, P., Hovmöller, S. & Abrahams, J. P. (2019). *ACS Cent. Sci.* **5**, 1315–1329. 10.1021/acscentsci.9b00394 PMC6716134 31482114

[bb17] Genderen, E. van, Clabbers, M. T. B., Das, P. P., Stewart, A., Nederlof, I., Barentsen, K. C., Portillo, Q., Pannu, N. S., Nicolopoulos, S., Gruene, T. & Abrahams, J. P. (2016). *Acta Cryst.* A**72**, 236–242. 10.1107/S2053273315022500 PMC4770873 26919375

[bb97] Guo, G., Fuchs, M. R., Shi, W., Skinner, J., Berman, E., Ogata, C. M., Hendrickson, W. A., McSweeney, S. & Liu, Q. (2018). *IUCrJ*, **5**, 238–246. 10.1107/S2052252518005389 PMC5929371 29755741

[bb18] Hattne, J., Echols, N., Tran, R., Kern, J., Gildea, R. J., Brewster, A. S., Alonso-Mori, R., Glöckner, C., Hellmich, J., Laksmono, H., Sierra, R. G., Lassalle-Kaiser, B., Lampe, A., Han, G., Gul, S., DiFiore, D., Milathianaki, D., Fry, A. R., Miahnahri, A., White, W. E., Schafer, D. W., Seibert, M. M., Koglin, J. E., Sokaras, D., Weng, T. C., Sellberg, J., Latimer, M. J., Glatzel, P., Zwart, P. H., Grosse-Kunstleve, R. W., Bogan, M. J., Messerschmidt, M., Williams, G. J., Boutet, S., Messinger, J., Zouni, A., Yano, J., Bergmann, U., Yachandra, V. K., Adams, P. D. & Sauter, N. K. (2014). *Nat. Methods*, **11**, 545–548.

[bb19] Hattne, J., Shi, D., de la Cruz, M. J., Reyes, F. E. & Gonen, T. (2016). *J. Appl. Cryst.* **49**, 1029–1034. 10.1107/S1600576716007196 PMC4886988 27275145

[bb20] Henderson, R. (1995). *Q. Rev. Biophys.* **28**, 171–193. 10.1017/s003358350000305x 7568675

[bb21] Kabsch, W. (2010). *Acta Cryst.* D**66**, 125–132. 10.1107/S0907444909047337 PMC2815665 20124692

[bb22] Kekilli, D., Moreno-Chicano, T., Chaplin, A. K., Horrell, S., Dworkowski, F. S. N., Worrall, J. A. R., Strange, R. W. & Hough, M. A. (2017). *IUCrJ*, **4**, 263–270. 10.1107/S2052252517002159 PMC5414400 28512573

[bb23] Leslie, A. G. W. (2006). *Acta Cryst.* D**62**, 48–57. 10.1107/S0907444905039107 16369093

[bb24] Li, X., Zhang, S., Zhang, J. & Sun, F. (2018). *Biophys. Rep.* **4**, 339–347. 10.1007/s41048-018-0075-x PMC6276065 30596142

[bb25] Martynowycz, M. W., Zhao, W., Hattne, J., Jensen, G. J. & Gonen, T. (2019). *Structure*, **27**, 1594–1600. 10.1016/j.str.2019.07.004 PMC7145226 31422911

[bb26] Nannenga, B. L., Shi, D., Leslie, A. G. W. & Gonen, T. (2014). *Nat. Methods*, **11**, 927–930. 10.1038/nmeth.3043 PMC4149488 25086503

[bb27] Nass, K., Meinhart, A., Barends, T. R. M., Foucar, L., Gorel, A., Aquila, A., Botha, S., Doak, R. B., Koglin, J., Liang, M., Shoeman, R. L., Williams, G., Boutet, S. & Schlichting, I. (2016). *IUCrJ*, **3**, 180–191. 10.1107/S2052252516002980 PMC4856140 27158504

[bb28] Palatinus, L., Brázda, P., Boullay, P., Perez, O., Klementová, M., Petit, S., Eigner, V., Zaarour, M. & Mintova, S. (2017). *Science*, **355**, 166–169. 10.1126/science.aak9652 28082587

[bb99] Prince, E. (2006). *International Tables for Crystallography*, Vol. C, 1st online ed. Chester: International Union of Crystallography.

[bb29] Shen, J., Wong, C. F., Subramaniam, S., Albright, T. A. & McCammon, J. A. (1990). *J. Comput. Chem.* **11**, 346–350.

[bb31] Smeets, S., Zou, X. & Wan, W. (2018). *J. Appl. Cryst.* **51**, 1262–1273. 10.1107/S1600576718009500 PMC6157704 30279637

[bb98] Smith, G. D., Pangborn, W. A. & Blessing, R. H. (2005). *Acta Cryst.* D**61**, 1476–1482. 10.1107/S0907444905025771 16239724

[bb32] Subramaniam, S., Faruqi, A. R., Oesterhelt, D. & Henderson, R. (1997). *Proc. Natl Acad. Sci. USA*, **94**, 1767–1772. 10.1073/pnas.94.5.1767 PMC19991 9050853

[bb33] Subramanian, G., Basu, S., Liu, H., Zuo, J.-M. & Spence, J. C. H. (2015). *Ultramicroscopy*, **148**, 87–93. 10.1016/j.ultramic.2014.08.013 25461585

[bb34] Takaba, K., Maki-Yonekura, S. & Yonekura, K. (2020). *J. Struct. Biol.* **211**, 107549. 10.1016/j.jsb.2020.107549 32544623

[bb35] Unwin, P. N. T. & Henderson, R. (1975). *J. Mol. Biol.* **94**, 425–440. 10.1016/0022-2836(75)90212-0 1236957

[bb36] Usón, I. & Sheldrick, G. M. (1999). *Curr. Opin. Struct. Biol.* **9**, 643–648. 10.1016/s0959-440x(99)00020-2 10508770

[bb37] Wang, B., Zou, X. & Smeets, S. (2019). *IUCrJ*, **6**, 854–867. 10.1107/S2052252519007681 PMC6760450 31576219

[bb38] Warshel, A., Sharma, P. K., Kato, M., Xiang, Y., Liu, H. & Olsson, M. H. M. (2006). *Chem. Rev.* **106**, 3210–3235. 10.1021/cr0503106 16895325

[bb39] Winn, M. D., Ballard, C. C., Cowtan, K. D., Dodson, E. J., Emsley, P., Evans, P. R., Keegan, R. M., Krissinel, E. B., Leslie, A. G. W., McCoy, A., McNicholas, S. J., Murshudov, G. N., Pannu, N. S., Potterton, E. A., Powell, H. R., Read, R. J., Vagin, A. & Wilson, K. S. (2011). *Acta Cryst.* D**67**, 235–242. 10.1107/S0907444910045749 PMC3069738 21460441

[bb41] Xu, H., Lebrette, H., Yang, T., Srinivas, V., Hovmöller, S., Högbom, M. & Zou, X. (2018). *Structure*, **26**, 667–675. 10.1016/j.str.2018.02.015 29551291

[bb42] Yonekura, K., Kato, K., Ogasawara, M., Tomita, M. & Toyoshima, C. (2015). *Proc. Natl Acad. Sci. USA*, **112**, 3368–3373. 10.1073/pnas.1500724112 PMC4372003 25730881

[bb43] Yonekura, K. & Maki-Yonekura, S. (2016). *J. Appl. Cryst.* **49**, 1517–1523.

[bb44] Zhou, H., Luo, Z. & Li, X. (2019). *J. Struct. Biol.* **205**, 59–64. 10.1016/j.jsb.2019.02.004 30794865

