# Supplementary material for: Statistically correcting dynamical electron scattering improves the refinement of protein nanocrystals, including charge refinement of coordinated metals
Source: Acta Crystallogr D Struct Biol. 2021 Jan 1;77(Pt 1):75–85. doi: 10.1107/S2059798320014540 (PMC7787111; doi:10.1107/S2059798320014540)
Supplement: Supplementary file 1 [file d-77-00075-sup1.pdf]

STRUCTURAL
BIOLOGY

Volume 76 (2020)

Supporting information for article:

Statistically correcting dynamical electron scattering improves refinement of protein nanocrystals, including charge refinement of coordinated metals

Thorsten B. Blum, Dominique Housset, Max T. B. Clabbers, Eric van Genderen, Maria Bacia-Verloop, Ulrich Zander, Andrew A. McCarthy, Guy Schoehn, Wai Li Ling and Jan Pieter Abrahams

Table S1 Insulin data acquisition and integration statistics of individual crystals used for data merging

	1	2	3	4	5	6	7
**Data collection**	(03)	(05)	(06)	(07)	(Ins-05)	(Ins-20)	(Ins-22)
Wavelength (Å)	0.02508						
Frame exposure (s)	0.6	1.5	1.2	1.2	0.6	0.6	0.6
Number of frames	50	60	65	60	80	70	90
Total expo-sure time (s)	30	90	78	72	48	42	54
Φ_{total} (˚)	9.3	19.1	16.6	21.9	30.2	26.5	34.0
$\Delta\varphi$ (˚/frame)	0.1855	0.3179	0.2556	0.3654	0.3779	0.3779	0.3779
Detector distance (mm)	900	900	900	900	1890	1890	1890
**Data processing**							
Space group	***R***3 (H3)	***R***3 (H3)	***R***3 (H3)	***R***3 (H3)	***R***3 (H3)	***R***3 (H3)	***R***3 (H3)
Unit cell dimensions							
a, b, c (Å)	a=b=82.54 c=33.50	a=b=82.56 c=33.51	a=b=82.41 c=33.15	a=b=82.40 c=33.46	a=b=82.65 c=33.67	a=b=83.05 c=33.64	a=b=82.46 c=34.02
α, β, γ (˚)	90, 90, 120	90, 90, 120	90, 90, 120	90, 90, 120	90, 90, 120	90, 90, 120	90, 90, 120
Resolution (Å)	22.6 – 3.53 (5.0 – 3.53)	17.1 – 3.12 (3.49 – 3.12)	23.8 – 3.12 (3.49 – 3.12)	20.60 – 3.02 (3.31 – 3.02)	24.53 – 3.30 (3.69 – 3.30)	21.14 – 3.17 (3.54 – 3.17)	30.73 – 3.67 (4.24 – 3.67)
R_{meas}	0.297 (0.313)	0.254 (0.645)	0.231 (0.619)	0.238 (0.704)	0.205 (0.882)	0.196 (1.04)	0.133 (0.477)
I/σI	3.63 (3.31)	3.33 (1.54)	3.55 (1.44)	3.88 (1.12)	3.11 (1.03)	11.2 (3.35)	4.47 (2.58)
Completeness (%)	14.6 (14.8)	30.9 (32.1)	25.2 (24.8)	34.2 (36.7)	38.4 (4.1)	34.0 (33.7)	40.0 (39.1)
Reflections	273 (183)	873 (264)	731 (213)	1034 (265)	616 (204)	599 (205)	479 (155)
Unique reflections	153 (100)	470 (139)	378 (107)	571 (149)	493 (153)	504 (147)	380 (131)
redundancy	1.78	1.86	1.93	1.81	1.25	1.19	1.26
R_{sym}	0.211 (0.222)	0.181 (0.457)	0.171 (0.457)	0.169 (0.499)	0.147 (0.624)	0.141 (0.743)	0.096 (0.337)
$CC_{1/2}$	0.946 (0.903)	0.964 (0.804)	0.970 (0.823)	0.955 (0.772)	0.976 (0.632)	0.994 (0.465)	0.987 (0.795)
Correlation with X-ray data (resol 50 – 4.0 Å)							
Indexation 1	0.77	0.56	0.65	0.70	0.66	0.74	0.65
Indexation 2	0.22	0.30	-0.03	0.06	0.03	0.03	0.14

Table S2 Thermolysin data acquisition and integration statistics of individual crystals used for data merging

**Data collection**	1	2
Wavelength (Å)	0.02508	0.02508
Frame exposure (s)	0.6	0.8
Number of frames	90	135
Total exposure time (s)	54	108
Φ_{total} (˚)	16.74	34.14
Δφ (˚/frame)	0.186	0.25289
Detector distance (mm)	1290.2	2345
Exposure dose (e.$Å^{-1}$.s^{-1})	0.0141	0.0141
**Data integration**		
Space group	$\boldsymbol{P}6_122$	$\boldsymbol{P}6_122$
Unit cell dimensions		
a, b, c (Å)	92.00, 92.00, 127.48	91.56, 91.56, 127.70
α, β, γ (˚)	90, 90, 120	90, 90, 120
Resolution (Å)	14.58-3.26 (3.34-3.26)	15.56-3.48 (3.57-3.48)
R_{meas}	0.472 (1.00)	0.745 (1.75)
I/σI	2.10 (1.08)	1.67 (1.01)
Completeness (%)	70.6 (71.6)	67.6 (68.8)
Reflections	9351 (679)	9447 (946)
Unique reflections	3800 (282)	2994 (220)

Table S3 Thaumatin data acquisition and integration statistics of individual crystals used for data merging

	1	2	3	4	5	6	7	8	9	10
**Data collection**	(21)	(33)	(19)	(30)	(27)	(08)	(14)	(16)	(10)	(09)
Wavelength (Å)	0.02508									
Frame exposure (s)	1.2	1.2	1.2	2.4	1.2	1.2	1.2	1.2	1.2	1.2
Number of frames	100	185	127	50	180	180	190	170	150	100
Total expo-sure time (s)	120	222	152.4	120	216	216	228	204	180	120
Φ_{total} (˚)	7.3	13.505	9.271	7.3	13.14	13.14	13.87	12.41	10.95	7.3
Δφ (˚/frame)	0.073	0.073	0.073	0.146	0.073	0.073	0.073	0.073	0.073	0.073
Detector distance (mm)	2345	2345	2345	2345	2345	2345	2345	2345	2345	2345
**Data integration**										
Space group	$P4_12_12$									
Unit cell dimensions										
a, b, c (Å)	57.72, 57.72, 149.17									
α, β, γ (˚)	90, 90, 90									
Resolution (Å)	10.69-2.76 (2.86-2.76)	11.69-3.24 (3.37-3.24)	8.83-3.12 (3.34-3.12)	9.81-3.10 (3.27-3.10)	11.74-3.39 (3.54-3.39)	12.26-3.40 (3.54-3.40)	12.01-3.33 (3.47-3.33)	10.66-3.77 (4.03-3.77)	10.49-3.16 (3.32-3.16)	8.630-3.26 (3.52-3.26)
R_{meas}	0.390 (0.998)	0.417 (0.948)	0.577 (1.21)	0.388 (1.06)	0.456 (0.954)	0.524 (1.10)	0.522 (1.11)	0.764 (1.08)	0.484 (1.20)	0.592 (1.85)
I/σ(I)	2.58 (1.10)	2.20 (1.06)	1.59 (1.01)	1.97 (1.02)	1.79 (1.05)	1.66 (1.00)	2.31 (1.09)	1.54 (1.02)	2.81 (1.12)	2.26 (1.08)
Completeness (%)	21.7 (21.9)	29.7 (31.5)	17.0 (19.5)	20.5 (24.1)	31.0 (35.0)	34.7 (38.7)	31.3 (35.9)	29.1 (32.8)	22.4 (22.7)	15.7 (17.5)
Reflections	2682 (311)	2834 (397)	2114 (489)	1690 (327)	2374 (358)	2214 (308)	2280 (344)	1542 (368)	2222 (376)	1333 (352)
Unique reflections	1517 (145)	1309 (151)	837 (166)	1029 (171)	1201 (153)	1329 (154)	1282 (163)	837 (166)	1064 (138)	681 (150)

Table S4 Parametrization of electron atomic scattering factors for Zn and Ca ions, using the 5 gaussians model. Parameters were fitted to data in the range $0.02 \leq \sin\theta / \lambda \leq 0.5$

	a1, b1	a2, b2	a3, b3	a4, b4	a5, b5	R_{scat} (%)
$Zn^{+0.5}$	2.91473<br>2.77406	2.83557<br>30.9254	4.13357<br>187.481	63.2726<br>3600.28	15.0154<br>885.604	0.18
$Zn^{+0.75}$	2.96853<br>2.86011	22.8485<br>919.772	3.0442<br>33.842	95.0852<br>3649.59	6.2312<br>207.334	0.20
Zn^{+1}	3.01042<br>2.92881	126.93<br>3682.27	30.7484<br>942.7	8.42183<br>220.231	3.2772<br>36.6377	0.21
Zn^{+2}	3.12034<br>3.12016	62.9056<br>1000.92	254.57<br>3764.19	17.7729<br>251.618	4.53073<br>47.6443	0.22
$Ca^{+0.5}$	2.84711<br>3.8095	3.7506<br>34.3279	63.0883<br>3527.94	5.76723<br>153.973	14.6981<br>836.477	0.20
$Ca^{+0.75}$	2.91939<br>3.92932	3.9753<br>37.149	7.29405<br>179.917	22.5015<br>882.291	94.8902<br>3595.47	0.22
Ca^{+1}	2.97364<br>4.01986	126.761<br>3645.44	4.13874<br>39.4108	30.4422<br>916.795	9.10789<br>200.748	0.23
Ca^{+2}	3.11407<br>4.25523	4.58655<br>46.2978	62.7958<br>997.095	254.521<br>3758.58	17.7083<br>249.738	0.23

$R_{scat} = 100 * \Sigma|F_{Int.Tables}(s) - F_{5G}(s)| / \Sigma |F_{int.Tables}(s)|$

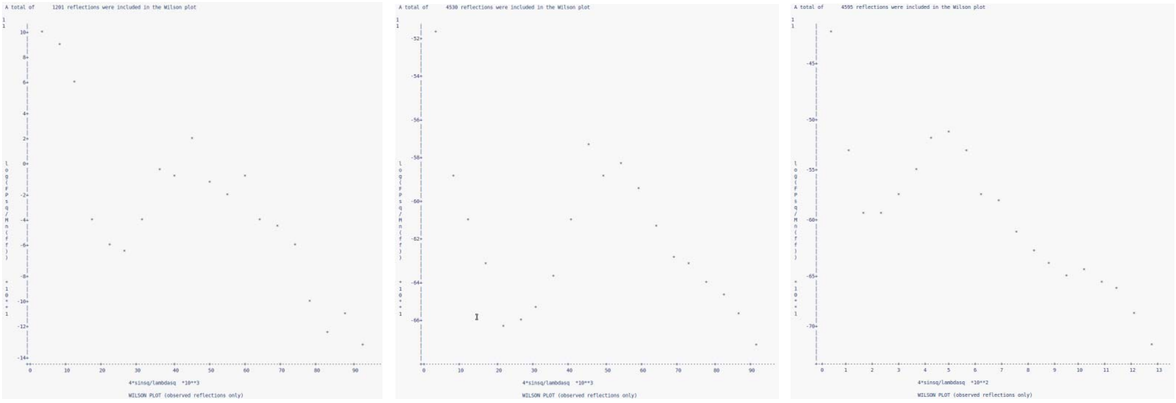

Insulin Thermolysin Thaumatin

Figure S1 Wilson plots of electron diffraction data for the three proteins.

S1. Data correction for dynamical scattering

S1.1. Theory

The likelihood-based correction described in (Clabbers et al., 2019) has been adapted in order to consider all measured intensities, including negative ones, in the calculation of the correction factor. The latter is calculated by comparing $I_o(\boldsymbol{h})$ versus $|F_c(\boldsymbol{h})|^2$ instead of $|F_o(\boldsymbol{h})|$ versus $|F_c(\boldsymbol{h})|$. The observed overestimation of weaker intensities $I_o(\boldsymbol{h})$ in comparison to squared calculated amplitude $|F_c(\boldsymbol{h})|^2$ has been attributed to a resolution dependent dynamical electron scattering term I_e, and we assumed the following approximation for the expected value of $I_o(\boldsymbol{h})$:

(1) $<I_o(h)> = (|F_c(h)|^4 + {I_e}^2)^{\frac{1}{2}}$

I_e is estimated by minimizing the sum within a chosen resolution bin:

(2) $\sum_h (I_o(h) - (|F_c(h)|^4 + {I_e}^2)^{\frac{1}{2}})^2$

The corrected intensity I_{corr}(h) is then provided by:

(3) $I_{corr}(h) = \frac{|F_c(h)|^2}{(|F_c(h)|^4 + I_e^2)^{\frac{1}{2}}} I_o(h)$ and $\sigma(I_{corr}(h)) = \frac{|F_c(h)|^2}{(|F_c(h)|^4 + {I_e}^2)^{\frac{1}{2}}} \sigma(I_o(h))$

S1.2. Methods

The three protein structures have been first refined against uncorrected structure factors using *REFMAC*. The final mtz file generated by *REFMAC* (refmac-out.mtz) contains the observed structure factors (F and SIGF), scaled to calculated structure factors (FC_ALL). In order to scale original intensities to the final calculated structure factors, one should first merge the original mtz file, original.mtz, containing unscaled IMEAN, SIGIMEAN, F, SIGF and the mtz file output by *REFMAC* (refmac-out.mtz) with *CAD* (ccp4 suite, (Winn et al., 2011)) and then do the scaling with *SCALEIT* (ccp4 suite). The following script will perform these two tasks (unscaled F and IMEAN are scaled to scaled observed structure factors provided by *REFMAC*):

```
#/bin/csh
```

```
#
cad HKLIN1 original.mtz HKLIN2 refmac-out.mtz.mtz HKLOUT cad-tmp.mtz > scale-
Imean.out << eof-cad
LABI FILE 1  E1=IMEAN E2=SIGIMEAN E3=F E4=SIGF E5=FreeR_flag
LABI FILE 2  E1=F E2=SIGF E3=FC_ALL E4=PHIC_ALL
LABO FILE 1  E1=IMEAN E2=SIGIMEAN E3=F E4=SIGF E5=FreeR_flag
LABO FILE 2  E1=FR E2=SIGFR E3=FC_ALL E4=PHIC_ALL
END
eof-cad
#
scaleit hklin cad-tmp.mtz hklout scaled-I.mtz >> scale-Imean.out << eof-sca
LABIN FP=FR SIGFP=SIGFR FPH1=F SIGFPH1=SIGF IMEAN1=IMEAN SIGIMEAN1=SIGIMEAN
REFINE ANISOTROPIC
NOWT
eof-sca
#
```

Then, the program *MTZ2VARIOUS* (ccp4 suite) can be used to produce an ascii file containing scaled IMEAN, SIGIMEAN and FC_ALL. These two columns can be imported in a spreadsheet software, such Microsoft Excel or LibreOffice. The use of the solver enables to determine I_e by minimizing $\sum_h (I_o(h) - (|F_c(h)|^4 + I_e{}^2)^{\frac{1}{2}})^2$. The corrected intensities $I_{corr}(\boldsymbol{h})$ can then be calculated using equation (3), either in the spreadsheet software or using the build-in functions and arithmetic operators available in *SFTOOLS* (ccp4 suite, (Winn et al., 2011)).
An example of *SFTOOLS* script is provided below (here I_e^2 = 142665):

```
#!/bin/bash
sftools << eof | tee sfcorr.log
READ scaled-I.mtz
CALC col A = col FC_ALL
CALC col B = col A col A *
CALC col C = col B col B * 142665 +
CALC col D = col C 0.5 **
CALC col E = col B col D /
CALC col IMEANcorr = col IMEAN col E *
CALC col SIGIMEANcorr = col SIGIMEAN col E *
DELETE col A
DELETE col B
```

```
DELETE col C
DELETE col D
DELETE col E
WRITE scaled-I-corrected.mtz
eof
```

References

Clabbers, M.T.B., Gruene, T., van Genderen, E., and Abrahams, J.P. (2019). *Acta Cryst.* A**75**, 82–93.

Winn, M.D., Ballard, C.C., Cowtan, K.D., Dodson, E.J., Emsley, P., Evans, P.R., Keegan, R.M., Krissinel, E.B., Leslie, A.G.W., McCoy, A., et al. (2011). *Acta Cryst.* A**67**, 235–242.
